# Diagnostic Performance of a Next-Generation Virtual/Augmented Reality Headset: A Pilot Study of Diverticulitis on CT

**DOI:** 10.1007/s10278-024-01292-7

**Published:** 2024-11-04

**Authors:** Paul M. Murphy, Julie Y. An, Luke M. Wojdyla, Adam C. Searleman, Aman Khurana, Thomas W. Loehfelm, Kathryn J. Fowler, Christopher A. Longhurst

**Affiliations:** 1 University of California-San Diego, 9500 Gilman Dr, La Jolla, CA 92093 USA; 2 Department of Radiology, University of California-San Diego, 200 W. Arbor Dr. MC 0834, San Diego, CA 92103 USA

**Keywords:** Virtual reality, Image quality, Diagnostic performance, Computed tomography

## Abstract

Next-generation virtual/augmented reality (VR/AR) headsets may rival the desktop computer systems that are approved for clinical interpretation of radiologic images, but require validation for high-resolution low-luminance diagnoses like diverticulitis. The primary aim of this study is to compare diagnostic performance for detecting diverticulitis on CT between radiologists using a headset versus a desktop. The secondary aim is to survey participating radiologists about the usage of both devices. This pilot study retrospectively included 110 patients (mean age 64 ± 14 years, 62 women) who had abdomen/pelvis CT scans for which the report mentioned the presence or absence of diverticulitis. Scans were dichotomized and matched by time, for a total of 55 cases with diverticulitis and 55 controls with no diverticulitis. Six radiologists were oriented to the VR/AR headset (Apple Vision Pro) and viewer app (Visage Ease VP) using ten scans. They each scored 100 unknown scans on a 6-level scale for diverticulitis (1 = no diverticulitis, 6 = diverticulitis) on the headset and then on a desktop. Time per case was recorded. Finally, they completed a survey using 5-level scales about the ease of use of the headset and viewer app (1 = difficult, 5 = easy), about their experience with the headset (1 = bad, 5 = good), and about their preference between devices (1 = desktop, 5 = headset). Summary statistics and multi-reader multi-case ROC curves were calculated. The AUC (and 95% confidence interval) for diverticulitis was 0.93 (0.88–0.97) with the headset and 0.94 (0.91–0.98) with the desktop (*p* = 0.40). The median (and first-third quartiles) of time per case was 57 (41–76) seconds for the headset and 31 (22–64) seconds for the desktop (*p* < 0.001). Average survey scores ranged from 3.3 to 5 for ease of use, from 3 to 4.7 for experience, and from 2.2 to 3.3 for preference. Diagnostic performance for detecting diverticulitis on CT was similar between the next-generation VR/AR headset and desktop. Ease of use, experience, and preference varied across different aspects of the devices and among radiologists.

## Background

Next-generation virtual/augmented reality (VR/AR) headsets may rival the desktop computer systems that are approved for evaluation of radiologic images. VR/AR headsets are advanced displays worn over the eyes, which create an immersive 3D experience by rendering virtual objects either into a virtual environment (VR) or into an augmented environment based on the user’s actual surroundings (AR) [1]. Headsets have been investigated for clinical applications, such as image-guided procedures, and, in a few studies, for diagnostic interpretation in place of desktop displays [2–5].

Guidelines for radiologic displays specify many factors, such as the number and spacing of pixels in the display, which relate to resolution [6–8]. Some headset displays now contain up to 23 million pixels at a 7.5 µm pitch [9], whereas 4K desktop monitors have only 8 million pixels at around a 200 µm pitch. However, each display is viewed from a different distance [10]. Thus, each has a different relation to the maximum resolution of human vision (2.5 line pairs per mm at 60 cm, or 50 pixels per degree) upon which guidelines are based [11]. Comparison of resolution is further complicated by optical distortion from headset lenses and by image interpolation during 3D rendering.

Guidelines also specify the brightness of the display, which is quantified in terms of luminance, and must be calibrated across a range. Organic light emitting diode (OLED) displays used in some headsets have high maximum luminance [12], but calibration to the DICOM grayscale display function (GSDF), which deviates from linearity at low luminance [13], may not be simple to verify due to the user interface of the headset. These aspects of displays are important to understand because they affect the visibility of imaging features and thus may impact diagnostic performance.

Therefore, the adequacy of headsets for visualization of high-resolution, low-luminance imaging features merits investigation. The approach of this study is to assess adequacy on the basis of diagnostic performance for CT abnormalities in this regime. One such abnormality is fat stranding, which represents edema within fine interstitial spaces of low-density adipose tissue [14], and thus appears on CT as high-resolution lines on a low-luminance background. One diagnosis that depends on fat stranding is diverticulitis, in which colonic outpouchings become inflamed, causing mesenteric edema and fat stranding, out of proportion to mural edema and wall thickening [15].

Other abnormalities would be less appropriate for this assessment. For instance, fractures involve bones that are high-density on CT, so they are often displayed at high luminance. Similarly, the highest-risk hepatic lesions can exceed 2 cm in size [16], so they may be visible at low resolution. The appearance of any abnormality can change based on zoom and window-level settings. However, with typical settings, diverticulitis is more appropriate to assess the high-resolution, low-luminance regime that poses the greatest challenge for displays.

The primary aim of this pilot study is to compare diagnostic performance for diverticulitis on CT between radiologists using a headset versus a desktop. The secondary aim of this study is to survey participating radiologists on the ease of use of the headset, experience with the headset, and preferences between headset and desktop for viewing CT scans and 3D reconstructions.

## Methods

### Patient Population

This pilot study was HIPAA-compliant and IRB-approved with a waiver of informed consent due to minimal risk (UCSD IRB #810017). The human subjects of the study were considered to be the patients whose imaging was evaluated, not the radiologists serving as readers, since the data collected pertained to the imaging and displays rather than the readers. The study retrospectively included 110 patients from a single large urban academic health system who had abdomen/pelvis CT scans with intravenous but not enteric contrast between 3/21/2023 and 1/24/2024 ordered by the emergency department (ED) due to pain, for which the impression of the report mentioned the presence or absence of diverticulitis. Using the report as reference standard, scans were dichotomized and matched by time, for a total of 55 cases with diverticulitis and 55 controls with no diverticulitis. Only one scan per patient was included. Ten scans were reserved for orientation to the appearance of diverticulitis, while 100 were used for a reader study. This number was chosen for feasibility since this was a pilot study, and sample size/power calculations could not be performed in the absence of preliminary data.

### Viewing Devices

**Fig. 1:**
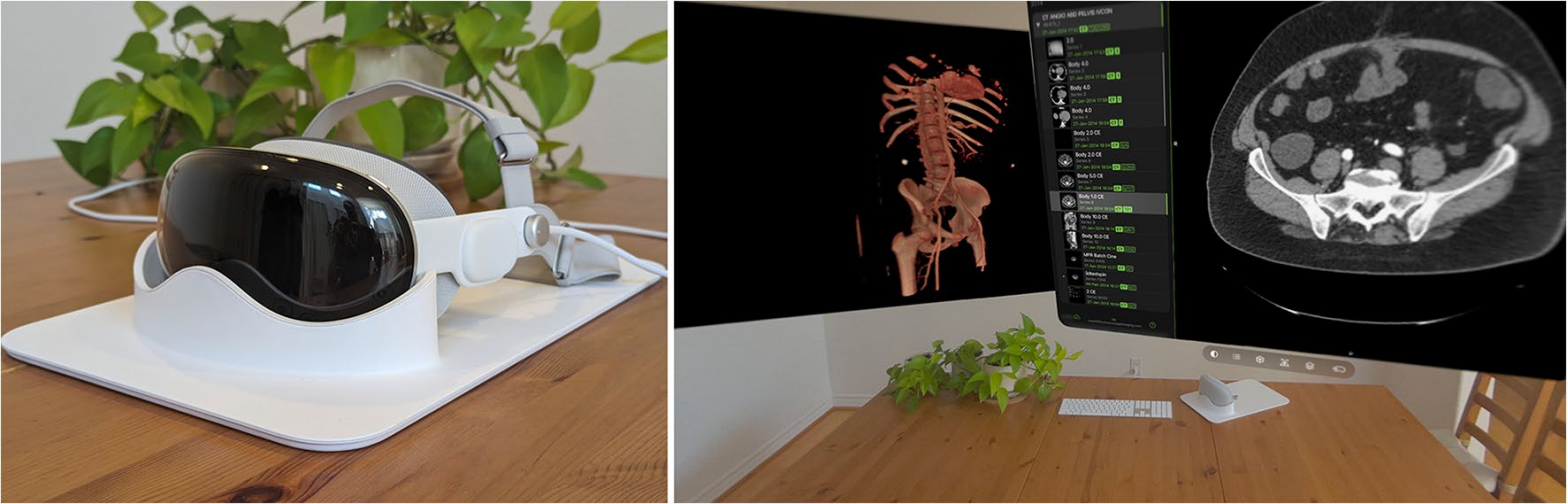
Virtual/augmented reality headset (left) and viewer app within the headset (right) illustrating a CT scan, 3D reconstruction, and list of images

Images were viewed in Visage Ease VP [17] on an Apple Vision Pro headset (Fig. 1) and in Visage 7 on a desktop diagnostic monitor. The luminance of the desktop had been DICOM GSDF calibrated for clinical use. No utilities were available to calibrate the luminance of the headset, so off-the-shelf settings were used instead. The Visage Ease VP app rendered cross-sectional images as though they were on a stationary flat screen within the augmented reality environment of the headset user. The app could also render reconstructions of bones and vessels as virtual 3D objects within the environment, though diagnostic performance for diverticulitis was assessed using only cross-sectional images and not 3D reconstructions.

For each reader, a feature of the Apple Vision Pro called “Guest User Mode” was used to calibrate the eye and hand tracking that serves as the user interface of the headset. Readers who needed them wore contact lenses while using the headset, as no optical inserts were available. Readers were oriented to the headset by performing several tasks pertinent to using the app, viewing CT scans, and viewing 3D reconstructions, as instructed by the study coordinator. Readers assessed the visibility of 5 and 95% luminance boxes and of 1- and 2-pixel line pairs of an 800 × 600-pixel TG18 test image [6], chosen due to similarity to the CT matrix size of 512 × 512 rather than the resolution of the headset display. Readers were seated during most of the assessment, since apps were stationary within the environment, but needed to rotate their heads to view different parts of different apps.

### Reader Study

**Fig. 2:**
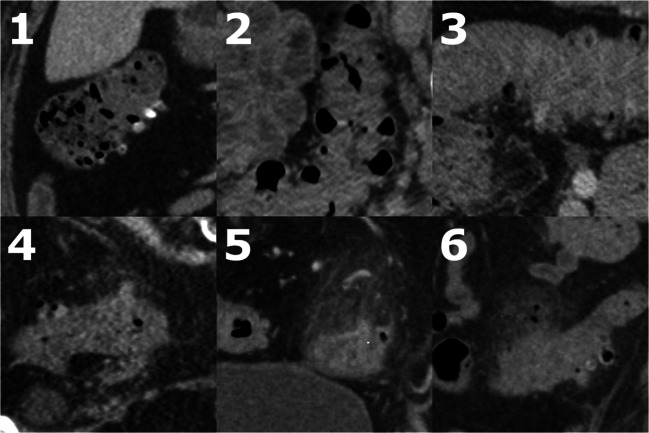
6-level scale used for diverticulitis, with examples from CT scans of this study. **1** Definitely no diverticulitis, e.g., diverticulosis with no wall thickening or fat standing. **2** Unlikely to be diverticulitis, e.g., diverticulosis with wall thickening but no fat stranding. **3** Indeterminate for diverticulitis, e.g., diverticulosis with a long segment of wall thickening and fat stranding. **4** Likely diverticulitis, e.g., diverticulosis with a short segment of wall thickening and mild fat stranding. **5** Definitely diverticulitis, e.g., diverticulosis with short segment of wall thickening and marked fat stranding. **6** Diverticulitis with perforation, e.g., same as 4 or 5 but with extraluminal gas or fluid collection

Two radiology residents and four body attendings with 4, 4, 6, 10, 13, and 18 years of experience participated as readers. Since readers were not considered human subjects of this study, other information about them, such as their need for contact lenses or their experience with other headsets, was not collected. Readers viewed five cases without diverticulitis and five cases with diverticulitis, to orient to their appearance on the headset. Readers then viewed 100 unknown cases and scored each on the 6-level scale for diverticulitis (Fig. 2). This scale was devised for this study based on features of diverticulitis as they relate to display quality since prior scoring systems relate to surgical management instead [18, 19]. Evaluation was performed using the headset during a single session of approximately 2 h duration and was repeated using a desktop in a different order after a 1-week washout period to minimize recollection. The number of cases, amount of work per case, and washout interval were chosen based on preliminary experience with the headset and cases. They were chosen to ensure that the duration of the session would be tolerable, though long enough to gain familiarity with the headset, and to ensure that recollection of cases would be minimal, given the simplicity and similarity of different diagnoses of diverticulitis. Readers were informed that each case was an ED patient presenting with abdominal pain, but further localizing information, clinical notes, and laboratory values were not available. Prior studies were available, but readers were instructed to score based on the current study. Scores were recorded in a spreadsheet on both headset and desktop. The time at which each score was recorded was captured. Time per case was calculated as the interval between scores.

### Survey

Readers were surveyed about the ease of use of the headset for each task performed during orientation, about their experience with three aspects of the headset, and about their preference between the headset and the desktop for three tasks. They scored each on a 5-level scale (Table 1).

**Table 1:** Survey scales for assessment of ease of use, user experience, and user preference. Readers scored the ease of use of the headset for several tasks pertinent to using the app, viewing CT scans, and viewing 3D reconstructions. Readers scored their user experience with several aspects of the headset and scored their preference between the headset and desktop for several activities

Score	Ease of use	User experience	User preference
1	Very difficult	Very bad	Strongly prefer desktop
2	Somewhat difficult	Somewhat bad	Somewhat prefer desktop
3	Neither	Neutral	No preference
4	Somewhat easy	Somewhat good	Somewhat prefer headset
5	Very easy	Very good	Strongly prefer headset

### Statistical Analysis

Summary statistics of the patient population and survey results were calculated and compared using *t*-tests and Fisher’s exact tests. Multi-reader multi-case ROC analysis was performed using the R package “MRMCaov” [20, 21]. AUCs were calculated by varying the threshold of the diverticulitis score, with the dichotomized diagnosis of the clinical report as a reference standard, and were compared between headset versus desktop. Time per case was compared using Wilcoxon’s signed rank test paired by reader and case. The normality of time per case was not presumed, due to outliers from readers taking breaks between some cases.

## Results

### Patient Population

In total, 110 patients were included with a mean age of 64 ± 14 years, with 62 females. The mean age was 63 ± 18 years for 10 patients in the orientation set, and 64 ± 14 years for 100 patients in the reader set (*p* = 0.43). The orientation set had 4 males and 6 females, and the reader set had 44 males and 56 females, with no association between set and sex (*p* = 0.74).

In the reader set, the mean age was 61 ± 16 for 44 males and 66 ± 11 years for 56 females (*p* = 0.03) and was 61 ± 13 for 50 patients with diverticulitis and 67 ± 14 years for 50 patients with no diverticulitis (*p* = 0.02). 20 males and 30 females had diverticulitis, and 24 males and 26 females had no diverticulitis, with no association between diagnosis and sex (*p* = 0.55).

### ROC Analysis

**Fig. 3:**
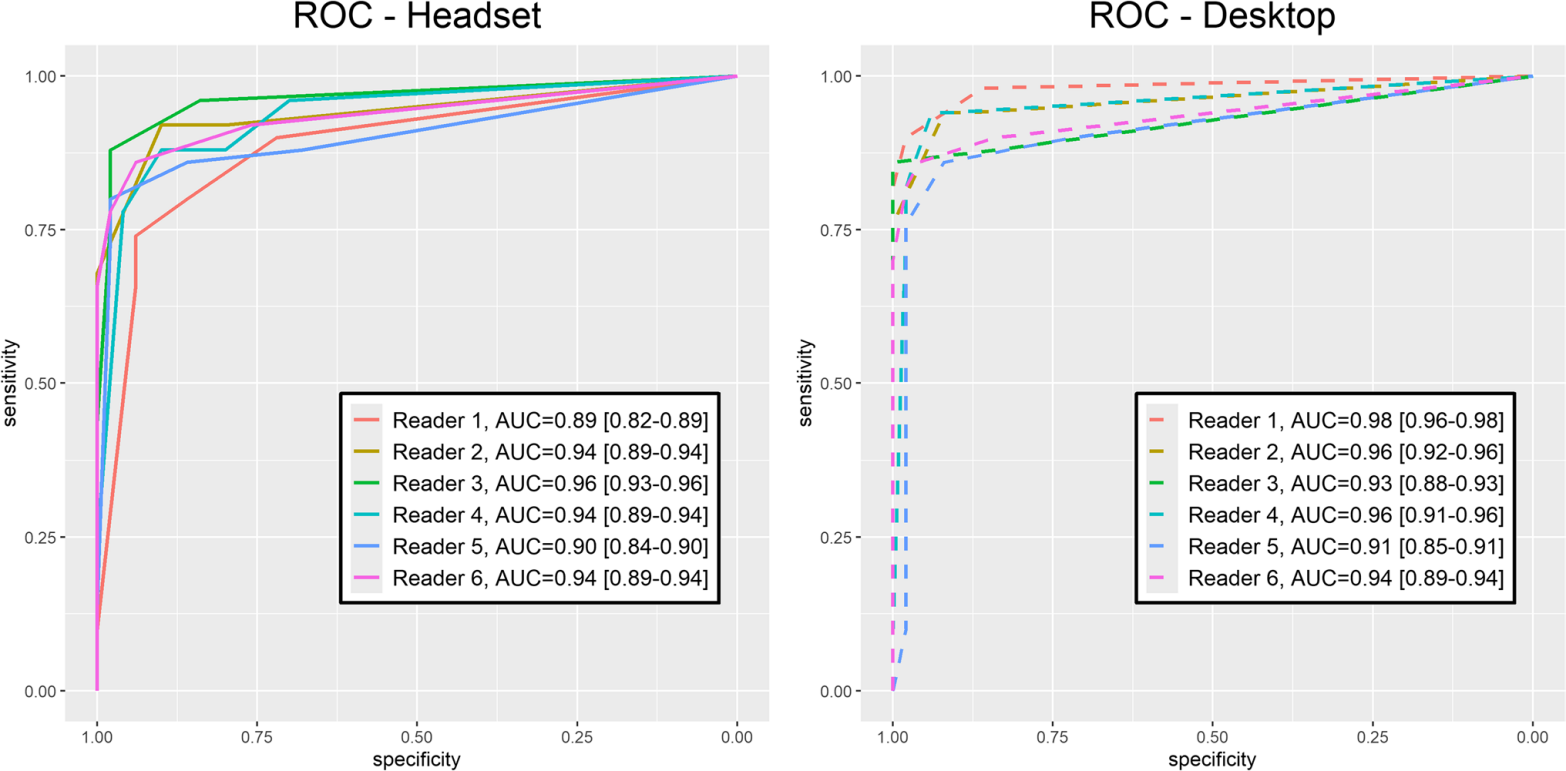
ROC curves and AUCs (with 95% confidence intervals) are shown for each reader for both headset (left) and desktop (right)

ROC curves and AUCs for each reader for both headset and desktop are shown (Fig. 3). Pooled AUC (and 95% confidence interval) was 0.93 (0.88–0.97) for the headset and 0.94 (0.91–0.98) for the desktop. This difference was not significant using random effects for readers (*p* = 0.40). This suggests diverticulitis could be adequately visualized on both devices for radiologists overall. Using fixed effects for readers, only one reader had a significantly different AUC between the headset and desktop (*p* < 0.01, others; *p* > 0.1). This study was not designed to investigate the cause of variation among readers. Imperfect agreement between scores and diagnoses may reflect additional information available only clinically, or diagnostic uncertainty since uncomplicated diverticulitis on imaging is seldom confirmed by surgery or pathology.

### Time Per Case

**Fig. 4:**
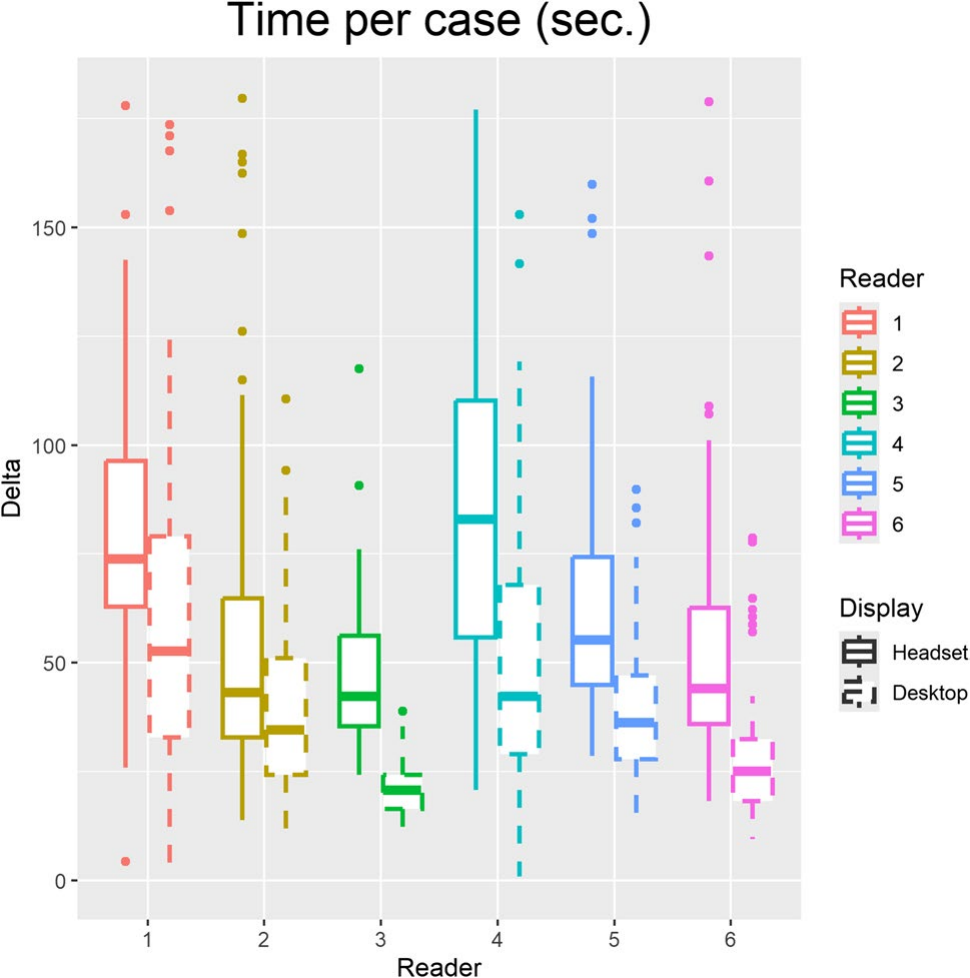
Boxplots illustrating median, quartiles, and outliers of time per case for 6 readers. Some outliers lie beyond the y-limit of this graph and are not shown

The median (and first-third quartiles) of time per case was 57 (41–76) seconds for the headset and 31 (22–64) seconds for the desktop (Fig. 4). These differences were significantly different (*p* < 0.001), for several possible reasons. Readers may have been more familiar with the desktop system, having used it professionally, whereas the headset had been released for only 3 months. Also, downloading cases to the headset over Wi-Fi may have taken longer than for the desktop over Ethernet.

### Survey

Survey results are shown (Table 2). Mean scores for ease of use of the headset ranged from 4.2 to 4.7 for basic app usage, from 3.7 to 5 for viewing CT scans, and from 3.3 to 4.7 for viewing 3D reconstructions. This indicates that most tasks were somewhat or very easy to perform on the headset.

**Table 2:** Survey scores for assessment of ease of use, user experience, and user preference

Task	Score			
	Mean	Stdev	Min	Max
**Ease of use (app)**				
Center the app window	4.3	0.5	4	5
Resize the app window	4.2	0.8	3	5
Open a list of studies	4.5	0.5	4	5
Open a study from a list	4.7	0.5	4	5
Close a study	4.7	0.8	3	5
**Ease of use (CT)**				
See a 5% luminance box	5	0	5	5
See a 95% luminance box	5	0	5	5
See the 1 pixel line pairs	4.8	0.4	4	5
See the 2 pixel line pairs	5	0	5	5
Window-level an image	3.8	1.3	2	5
Zoom into an image	4	0.9	3	5
Scroll through images	3.7	1.4	2	5
**Ease of use (3D)**				
Open a 3D recon	4.7	0.5	4	5
Center the 3D recon	4.2	1	3	5
Reorient the 3D recon	4	1.3	2	5
Window-level a 3D recon	3.3	1	2	5
Crop a 3D recon	3.7	0.8	3	5
**User experience**				
Motion sickness	4.7	0.8	3	5
Physical comfort	3	1.3	1	4
Eye and hand controls	4	0.6	3	5
**User preferences**				
Open a study from a list	2.2	1.2	1	4
View CT images	2.2	1.6	1	5
View 3D recons	3.3	1.4	1	5

The mean scores for user experience with the headset ranged from 3 to 4.7. The mean score for “motion sickness” was 4.7, indicating that the headset caused little motion sickness during use. The mean score for “physical comfort” was 3 but ranged from 1 to 5 among radiologists. This indicates the headset was very comfortable to some but very uncomfortable to others, during approximately 2 h of evaluation. Nonetheless, all 6 readers completed all 100 cases without needing to abort due to discomfort. The mean score for “eye and hand controls” was 4, indicating a somewhat good experience with the user interface. This is notable given the novelty of the eye tracking and hand gesture recognition of the headset.

The mean scores for user preference ranged from 2.2 to 3.3. The mean scores for “opening study from a list” and “viewing CT images” were both 2.2, indicating that radiologists somewhat preferred the desktop. The mean score for “viewing 3D reconstructions” was 3.3, indicating that radiologists somewhat preferred the headset. This suggests the immersive 3D experience of the headset surpasses the desktop. However, each of these scores ranged widely from 1 to 4 or 5 among radiologists, indicating that individual preferences are important to consider.

## Discussion

This pilot study demonstrated no significant difference in the pooled diagnostic performance for diverticulitis between radiologists using a headset versus a desktop. Diagnosis of diverticulitis on CT requires visualization of fat stranding, which presents a challenge for radiologic displays. This result suggests that the headset may suffice to display such high-resolution low-luminance features and provides preliminary data needed to design future reader studies on this topic. In addition, radiologists reported that most tasks were easy to perform using the headset (Table 2). User experience with the headset was good overall but varied among radiologists, as did preferences between headset and desktop, with the headset preferred only for viewing 3D reconstructions during orientation prior to the reader study. Overall, these results suggest that next-generation VR/AR headsets, with high-resolution displays that have been recently developed by vendors, offer promising functionality for viewing CT scans and 3D reconstructions.

The general results of this study are similar to prior investigations of virtual reality displays for medical imaging. These showed promising diagnostic performance of a headset for pulmonary nodules [5], favorable user experiences with a virtual reading room for presurgical planning [4], and many other potential applications such as intraoperative guidance, where augmented reality may become essential [1–3]. Eye tracking has been investigated extensively in radiology, for studying search patterns [22, 23] and accelerating image annotation [24–28]. These utilized desktop-based devices, but headset-based devices may facilitate eye tracking in additional applications. Lastly, the comparison of diagnostic performance is limited since the scale used for diverticulitis was devised for this study. Other classification systems such as Hinchey, Modified Hinchey, or AAST [18, 19] were not appropriate, since they focus on features of complicated diverticulitis that guide surgical management, rather than features of uncomplicated diverticulitis that relate to display quality.

This study has several limitations. First, it was not IRB-approved or statistically powered to investigate whether factors relating to the readers impacted any measured outcome. Future studies with a larger number of readers may be required to explain variations in performance and preference among individual radiologists. For instance, comparing contact lenses versus optical inserts available from vendors may be important for users needing vision correction. Second, although fat stranding is an important feature of diverticulitis, other lower-resolution higher-luminance features, such as colonic wall thickening, also contribute. Furthermore, uncomplicated diverticulitis is seldom confirmed surgically or pathologically. Thus, diverticulitis on CT reports may not perfectly assess display quality. Other diagnoses, such as subtle hepatic lesions or non-displaced fractures, may be considered for future studies. Third, reader sessions with the headset always preceded the desktop due to scheduling constraints for the orientation and sessions. This could have biased results in favor of the desktop, for instance, by contributing to a lower time per case; however, no effect on pooled diagnostic performance was seen. Lastly, technical aspects of this and other headsets, such as their actual resolution and luminance, were not directly measured. This study was only intended to examine their diagnostic impact for one headset, and other headsets were not compared. Such measurements may require collaboration between vendors and professional societies. Other radiology workflows such as reporting would also require vendor collaboration and design consideration to migrate to the headset.

Usage of the term “resolution” differs between image display and image acquisition [29]. The resolution of image displays is often used to mean screen size in units of pixels. The resolution of image acquisitions means the smallest distance at which objects can be distinguished and is similar to the voxel size of an acquisition. The matrix size of an acquisition is more comparable to the screen size of a display. These usages are related since a higher-resolution acquisition would require a higher-resolution display if every acquired voxel is to be displayed without minification for a given field of view. Fat stranding is a high-resolution feature in the latter sense, since it occurs over small distances within the field-of-view. However, fat stranding may not be a high-resolution feature of the image display in the former sense, since acquired pixels of the CT may be magnified across multiple display pixels. Higher matrix size modalities like radiography may present additional challenges for image displays.

## Conclusion

Given the importance of CT within radiology, it is important to address whether its highest-resolution features can be visualized on imaging displays such as those of next-generation VR/AR headsets. This pilot study found no significant difference in the pooled diagnostic performance for diverticulitis between radiologists using a headset versus a desktop, and radiologists reported an overall good user experience with the headset. These results suggest that the display of this headset may suffice for visualization of such features. In addition, the headset offers new opportunities for 3D visualization through its immersive display and new possibilities for ergonomics through the eye and hand tracking of its user interface. For these reasons, next-generation VR/AR headsets are promising for many applications throughout radiology.

## Data Availability

Data generated or analyzed during the study are available from the corresponding author by request.
